# Hybrid Nanoparticles at Fluid–Fluid Interfaces: Insight from Theory and Simulation

**DOI:** 10.3390/ijms24054564

**Published:** 2023-02-26

**Authors:** Małgorzata Borówko, Tomasz Staszewski

**Affiliations:** Department of Theoretical Chemistry, Institute of Chemical Sciences, Faculty of Chemistry, Maria Curie-Skłodowska University, 20-031 Lublin, Poland

**Keywords:** particle-laden interfaces, Janus particles, ligand-tethered particles, molecular simulations

## Abstract

Hybrid nanoparticles that combine special properties of their different parts have numerous applications in electronics, optics, catalysis, medicine, and many others. Of the currently produced particles, Janus particles and ligand-tethered (hairy) particles are of particular interest both from a practical and purely cognitive point of view. Understanding their behavior at fluid interfaces is important to many fields because particle-laden interfaces are ubiquitous in nature and industry. We provide a review of the literature, focusing on theoretical studies of hybrid particles at fluid–fluid interfaces. Our goal is to give a link between simple phenomenological models and advanced molecular simulations. We analyze the adsorption of individual Janus particles and hairy particles at the interfaces. Then, their interfacial assembly is also discussed. The simple equations for the attachment energy of various Janus particles are presented. We discuss how such parameters as the particle size, the particle shape, the relative sizes of different patches, and the amphiphilicity affect particle adsorption. This is essential for taking advantage of the particle capacity to stabilize interfaces. Representative examples of molecular simulations were presented. We show that the simple models surprisingly well reproduce experimental and simulation data. In the case of hairy particles, we concentrate on the effects of reconfiguration of the polymer brushes at the interface. This review is expected to provide a general perspective on the subject and may be helpful to many researchers and technologists working with particle-laden layers.

## 1. Introduction

In recent years, there has been an increasing interest in the investigation of nanoparticles adsorbed at fluid interfaces [1,2,3,4,5,6,7,8,9,10,11,12]. The fluid interfaces provide a suitable environment for the assembly of particles into structures with technological applications in various fields. The particle-laden interfaces can be used as support of novel applications which range from the stabilization of dispersed systems, including emulsions, foams, colloidosomes, liquid marbles, the production of functional materials with unique electrical, optical, or magnetic properties, and enhanced oil recovery [7,8,9,10,11,13,14,15,16,17,18]. Nanoparticles at liquid–liquid interfaces are also used for enhanced biphasic catalysis [19]. The behavior of nanoparticles at fluid–fluid interfaces is also very interesting from a purely cognitive point of view. The particle-laden layers can be used to study some basic problems in condensed matter physics. The particles are confined into a quasi-two-dimensional interface between two fluids. This leads to the emergence of completely new behaviors and properties that are not observed in the three-dimensional systems, for example, fascinating two-dimensional phase transitions or anomalous rheological responses [3]. The theoretical description of such systems requires considering forces acting at the molecular scale with mechanical ones operating at the microscale or even larger distances [1,12].

The behavior of different nanofluids has been recently studied [20,21,22,23,24,25]. However, nanoparticles at interfaces are of particular interest. The development of the synthesis method has enabled the production of various particles that differ in shape, size, or surface chemistry. These particles can have sizes ranging from a few nanometers to several micrometers. It is possible to fabricate particles of various shapes, for example, spherical, ellipsoidal, cuboidal, cylindrical, and many others, including exotic two-dimensional structures [2,13,14]. Furthermore, the surface of the particles can be modified in different ways. The surface patterned particles contain domains (patches) with different chemical or physical properties. Among the patchy particles, so-called Janus particles with two distinct parts seem to be the most popular. The interest in exploiting Janus particles for stabilizing interfaces originated from the Nobel Lecture of Pierre-Gilles de Gennes, in which their use was proposed to replace molecular surfactants [26]. Now, Janus particles have numerous practical applications, and they are used as building blocks in the bottom-up assembly of novel materials [27,28,29,30,31], as active nanomotors [32,33,34,35,36,37,38,39], for the stabilization of emulsions [40], foams, and polymer blends [41,42,43,44,45,46,47,48], in biological imaging and sensing [49,50], and in drug delivery [51,52,53]. Quite recently, the application of Janus particles in enhanced oil recovery processes was reviewed [54,55].

The interfacial applications are of particular importance since Janus particles exhibit a large surface activity. These particles combine the colloidal-scale properties of particle stabilizers (large energy of desorption from fluid interfaces) with the molecular-scale properties of surfactants (amphiphilicity and reduction of interfacial tension) [3].

Another important group of particles with a modified surface are “hairy” particles [56,57]. Their surfaces are covered by a layer of organic ligands. Hairy particles simultaneously have the physical (optical, electronic, magnetic) properties of the inorganic core and the mechanical strength, flexibility, processability, and dielectric properties of the organic coatings. Due to the presence of a soft organic canopy, these particles exhibit special properties. The shape of the hairy particles varies in a response to changes in the surrounding medium. Ligand-tethered nanoparticles are used to produce nanocomposites, sensors, drug delivery systems, and many other applications [53,56,57,58]. Biological and medical applications of hairy particles require an understanding of their behavior at interfaces and the transfer to cells. In this context, the study of hairy particles trapped at a fluid–fluid interface is one of the most important topics in colloid and surface science.

Janus particles and hairy particles are hybrid objects that combine different properties of their internal parts, which causes their unique behavior under certain conditions. These particles were the subject of numerous experimental and theoretical studies that were summarized in several reviews [1,2,3,4,5,6,10,11,13,14,59,60,61].

In this article, we focus on the behavior of Janus particles and hairy particles at the fluid–fluid interfaces. The purpose of this review is to describe and contextualize, from the author’s viewpoint, the representative theoretical works on the fluid–fluid interfaces, involving homogeneous particles, Janus particles, and hairy particles. We develop the link between simple phenomenological approaches and molecular simulation studies. We consider only the properties of the particle-laden layers at equilibrium. Readers interested in other issues, such as adsorption dynamics, particle transport, or kinetics, will be referred to the relevant articles.

The paper is organized as follows. In the next section, we analyze selected works devoted to chemically homogeneous particles of different shapes at fluid–fluid interfaces. We discuss, here, fundamental theoretical concepts and define the basic quantities characterizing the systems under consideration. Then, the most popular, idealized models are discussed and compared with the molecular simulations. The limitations of phenomenological approaches are signalized. A brief summary of research on the self-assembly of homogeneous particles ends this section. In Section 3, we focus on the behavior of different, hard Janus particles at the fluid–fluid interface and report the results obtained from the theory and simulations. Section 4 contains the analysis of representative works associated with the adsorption of polymer-tethered particles at fluid–fluid interfaces. We pay attention to the reconfiguration of the hairy particles at the interface and their assembly. The last section lists the issues discussed with the relevant literature references and general conclusions.

## 2. Chemically Homogeneous Particles at Fluid–Fluid Interfaces

### 2.1. Simple Phenomenological Models of Nanoparticles at Interfaces

The formation of any particle-laden fluid interface results from two processes: (i) the transport of particles to the interface and (ii) the breach of the interface as a result of particle protrusion [3]. In this work, we focus solely on the second process, i.e., the equilibrium adsorption of particles at the interface. The particle adsorption is associated with the reduction of the contact area between the two fluid phases, which leads to a decrease in the total free energy of the system. This is the result of competition between different interactions (enthalpy) and entropy effects.

The properties of particle-laden layers at the equilibrium depend on the nature of the interface, the particle-interface interactions, and inter-particle interactions. To predict the adsorption equilibrium, one should take into account the van der Waals and electrostatic interactions between all species, the interactions induced by the interface, such as the electrical double layer forces, capillary interactions, hydrodynamic interactions [62], and sometimes, also, externally actuated interactions. The interactions can be additionally modulated by the environmental conditions, for example pH, ionic strength, or temperature. This issue has been discussed in detail in the review [3]. The interactions between a solid particle and both fluids determine its wettabilities characterized by the contact angles. The wettability of nanoparticles can be adjusted in a wide range by modifying the surface [63].

Phenomena occurring at the fluid–fluid interface depend significantly on temperature. First of all, a stable fluid–fluid interface exists only in the limited ranges of temperatures. Moreover, the entrapment of a particle at a fluid interface is only possible when the difference between the energies of the particle dispersed in one of the bulk phases and that of the adsorbed particle exceeds the energy associated with thermal agitation. Similarly, the self-assembly of particles can be controlled by temperature. The impact of the temperature of interactions in biological fluids was analyzed by Mahmoudi et al. [64].

Let us now turn to a brief discussion of the thermodynamics of particle-laden phases. Thermodynamic models, employing the standard thermodynamic bulk quantities and parameters associated with the presence of interface (surface and line tensions), provide a powerful and simple theoretical background to understand the behavior of particles at fluid–fluid interfaces. We begin with the presentation of the theoretical basis for a particle with a homogeneous surface. The free energy of a system consisting of a nanoparticle (P) adsorbed at a planar interface that separates two fluids, W (polar) and O (apolar), is given by:

(1) $$F_{I}=F_{I}\left(X,S_{OW},S_{O},S_{W},L\right),$$

where $X=(V,T,N_{0},N_{W}),V$ is the volume of the system, *T* denotes temperature, $N_{j}$ is the number of particles of the jth fluid $\left(j=O,W\right),S_{j}$ is the area of the interface between the particle and the jth fluid, and $S_{OW}$ is the area of the O/W interface, while L denotes the length of three-phase contact line, where the particle and the two fluid phases meet [65].

The surface tension, $\gamma_{i}$ (associated with the interface between particle and fluid *j*), and the line tension, τ (associated with the particle–fluid–fluid contact line), are defined as [1]

(2) $$\gamma_{j}={\left(\frac{\partial F}{\partial S_{j}}\right)}_{X,S_{OW},S_{i\neq j}},$$

(3) $$\tau ={\left(\frac{\partial F}{\partial L}\right)}_{X,S_{OW},S_{O},S_{W}},$$

**Figure 1:**
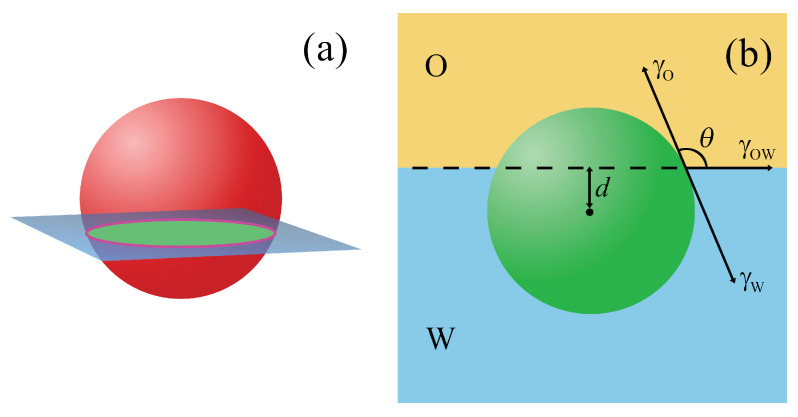
A spherical particle at a fluid/fluid interface (O/W). (**a**) The area of the fluid interface that is removed upon adsorption of the particle (green area) and the three-phase contact line associated with the line tension (magenta line). (**b**) The interface tension vectors, $\gamma_{O}$ and $\gamma_{W}$, are the interface tensions between the particle and the fluids, and $\gamma_{OW}$ is the interfacial tension corresponding to the fluid interface. *R* denotes the particle radius, θ is the contact angle, and *d* is the a position of the particle center.

The surface and line tensions are a result of the imbalance of intermolecular forces in the interfacial region and at the particle–fluid–fluid contact line, respectively (see Figure 1).

The free energy of the nanoparticle at the interface, $F_{I}$, is given by

(4) $$F_{I}=\gamma_{O}S_{O}+\gamma_{W}S_{W}+\gamma_{OW}S_{OW}+L\tau ,$$

where $S_{OW}=S_{I}-S_{r}$ and $S_{I}$ denotes the area of the interface without adsorbed particle, while $S_{r}$ is the surface occupied (removed) by a particle at the interface. Moreover, *S* is the total surface of the particle, $S=S_{O}+S_{W}$.

The interfacial energy is usually expressed with reference to the free energy of the particle immersed in one of the phases, $F_{jb}=F_{jb}\left(X,S_{O},S_{W}\right)$. A change in the free energy of a particle associated with the transfer from a single phase “*j*” to a flat O/W interface is given by

(5) $$\Delta F_{Ij}=F_{I}-F_{jb}=\pm \left(\gamma_{O}-\gamma_{W}\right)\left(S-S_{j}\right)-\gamma_{OW}S_{r}+\tau L,$$

the sign “+ “ (“−”) before the bracket corresponds to the transfer from W (from O). Equation (5) is general, and it can be used to study nanoparticles of different shapes. The attachment (trapping) free energy of a particle, ΔF, is equal to the lower of $\Delta F_{IO}$ and $\Delta F_{IW}$.

Most works involve the simplistic model [66], in which a particle is treated as a smooth, hard object, and the following assumptions are introduced: (i) gravitational forces are negligible, (ii) the energy contribution from the line tension is omitted (τL tends to zero), (iii) the capillary effects and interface deformations do not be taken into account, (iv) changes in entropy effects associated with movement from a three-dimensional to a two-dimensional environment is ignored, and (v) the electrostatic interactions are not explicitly involved.

**Figure 2:**
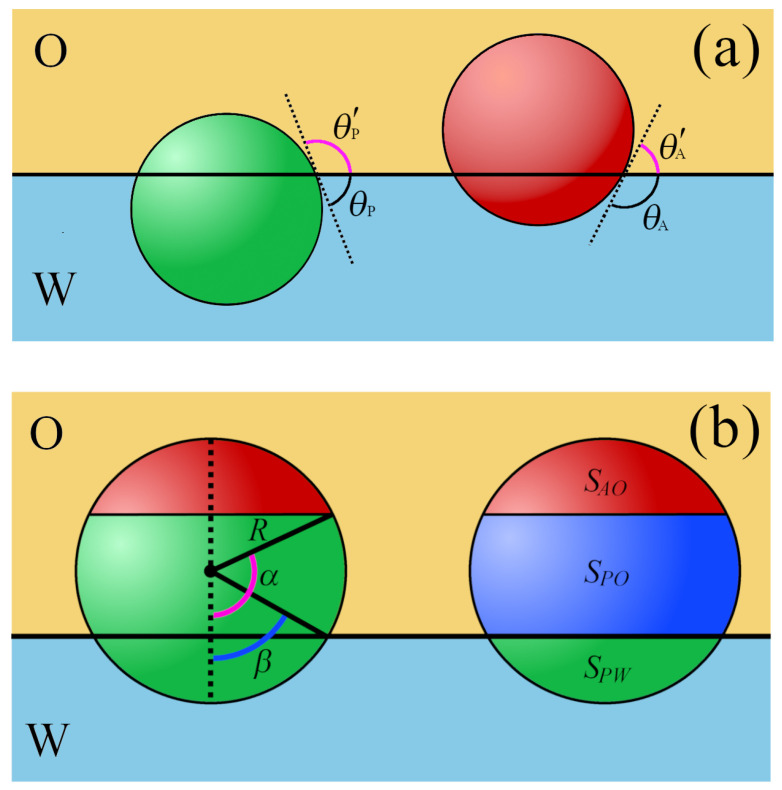
Spherical particles at a fluid/fluid interface (O/W). (**a**) The contact angles of homogeneous polar, $\theta_{P}$, and apolar, $\theta_{A}$, particles. (**b**) The left part: the geometry of a Janus particle: The relative areas of the polar (green) and apolar (red) particle patches are characterized by angle α and the contact angle of the Janus particle at interface β. The right part: areas of different particle–fluid interfaces, $S_{PW}$ for a polar region in contact with the fluid W (green), $S_{PO}$ for a polar region in contact with the fluid O (navy), and $S_{AW}$ for an apolar region in contact with the fluid O (red).

A degree of polarity of the particle surface can be characterized by the three-phase contact angle of the particle. In the literature, two definitions of the contact angle are used, θ [3] and $\theta \prime =180^{\circ }-\theta$ [67], as shown in Figure 2a. The contact angle θ is the angle between the plane tangent to the particle’s surface and the interface at the line where the interface meets the particle [3]. In the case of perfect polar (hydrophilic) and apolar (hydrophobic) surfaces, $\theta =180^{\circ }$ ($\theta \prime =0^{\circ }$) and $\theta =0^{\circ }$ ($\theta \prime =180^{\circ }$), respectively.

Then, from the Young equation, we have

(6) $$\cos\theta =-\cos\theta^{\prime }=\left(\gamma_{W}-\gamma_{O}\right)/\gamma_{OW},$$

Finally, in the framework of Pierański model [66], the change free energy associated with the transfer of a particle from the bulk phase “j” to the interface is given by

(7) $$\Delta E_{Ij}=\gamma_{OW}\left(\pm \left(S-S_{j}\right)\cos\theta -S_{r}\right),$$

with “+” for j=O and “−” for j=W.

The limitations of the above approach are discussed in numerous reviews [1,2,3,4,5,6,60]. We mention here some effects, which are neglected and can result in deviation from the simple model. First of all, line tension can play a considerable role in particle adsorption at a fluid–fluid interface. The impact of line tension was intensively investigated using various theoretical and experimental methods [68,69]. On theoretical grounds, the line tension can be either positive or negative, and it is expected to be a small force [65]. The contact line scales linearly with a radius of spherical particle (*R*), while its total interfacial area scales with $R^{2}$. Thus, line tension effects are more significant for small particles. Aveyard and co-workers [69] analyzed the effects of line tension on the adsorption free energy for the case of a smooth sphere. They showed that, for positive line tension, there is a tendency to reduce the length of the contact line, while, for negative line tension, the inverse effect is observed, and the contact line is maximized. Moreover, positive line tension leads to significant energy barriers and, in certain cases, may result in multiple energy minima in the interfacial free energy [69,70]. Cheung [71] carried out molecular dynamics simulations for spherical particles, taking into account the line tension. His study confirmed the reasonableness of the Pierański model. However, it was also shown that, for small particles, the incorporation of capillary waves into the predicted effective nanoparticle–interface interaction improves agreement between simulation and theory. In the case of non-spherical particles, the effect of line tension becomes more important as the contact line is often large relative to the case of a sphere.

As particle size decreases the assumption of a planar interface becomes increasingly invalid. For example, the description of adsorption on emulsion droplets should take the curvature into account. However, it has been theoretically proved that the curvature significantly affects the adsorption energy only when the radius of curvature is similar to the radius of the particle. Thus, in most typical experimental systems, it can be neglected [69].

Other significant effects can be associated with capillary waves and interface deformations. In the case of small particles (similar to fluid molecules), the assumption of a flat interface breaks down as capillary waves and individual molecular interactions result in an uneven interface. Such an interface is difficult to model using the simple model. Theoretical approaches and molecular simulations have shown that capillary waves result in a broadening of the adsorption energy well with respect to the flat interface model [72]. Due to capillary forces, adsorption of particles can change the shape of an initially flat fluid–fluid interface. Significant interfacial deformation can occur upon adsorption of non-spherical factors [73]. However, for an individual spherical particle, such deformation has little effect.

Furthermore, the particle roughness results in a large increase of the surface area in contact with the liquid phase and changes significantly the wettability of the particle [60,74,75,76]. Nonomura et al. [74] showed that the effect of increasing surface roughness was to accentuate the natural wettability of the particles, which weakened their activity as emulsifiers compared to a smooth analog. On the other hand, for particles with nanoscale roughness, an increase in the interfacial activity was also observed [76].

The transfer of a particle from a bulk phase to the interface is associated with a change in entropy. The estimation of the entropy change is difficult. Theoretical results of Aveyard et al. [77] suggested that this effect is negligible and can be reasonably ignored at the single particle level. Nevertheless, entropic effects can be significant for certain systems. When one of the liquid phases contains polymers, the migration of particles from this phase to the interfacial region can be entropically favorable. In this case, the increased freedom of the polymer chains upon demixing the colloid is sufficient to overcome the entropic penalty of placing the particles at the interface [60].

Additionally, electrostatic interactions play a significant role at numerous fluid–fluid interfaces and should be involved in more sophisticated approaches [3,78]. It is experimentally established that air–water or oil–water interfaces are negatively charged [78]. This means that, depending on the ionic strength, negative particles repelled by the interface adsorb either very slowly or not at all, whereas positively charged particles adsorb readily. In this way, the energy barrier is created on the water side of the interface (Figure 1 in [78]).

Quite recently, Vialetto et al. [79] presented a review on phenomena involving the attachment and detachment of colloidal particles to and from fluid interfaces.

#### 2.1.1. Spherical Particles with Homogeneous Surfaces

In the case of a homogeneous spherical particle of the radius *R*, we can put $S_{O}=2\pi r\left(R-d\right)$ and $S_{r}=\pi \left(R^{2}-d^{2}\right)$ to Equation (7). Then, a change in the free energy associated with the particle transfer from a point located at a depth z=d in the bulk W to the interface can be calculated. Finally, a quadratic function ΔE(z) is obtained (see Figure 2 in [60])

(8) $$\frac{\Delta E_{Ij}}{\pi \gamma_{OW}}=z^{2}+\bar{b}z+\bar{c},$$

where $\bar{b}=-2R\left(\gamma_{O}-\gamma_{W}\right)/\gamma_{WO}$,  $\bar{c}=-2R^{2}\left(\gamma_{O}-\gamma_{W}\right)/\gamma_{WO}-R^{2}$ and −R≤z<0 (with the interface located at z=0). In the same way, the transfer energy from the bulk phase O can be calculated.

The equilibrium position of the particle corresponds to a minimum of this function

(9) $$d=z_{O}=R\left(\gamma_{O}-\gamma_{W}\right)/\gamma_{OW},$$

The normalized position of spherical particles (d′=d/R) in relation to the interfacial plane can be defined exclusively by the contact angle, θ (compare Equations (6) and (9)).

Inserting this value into Equation (8), we obtain the well known expression for the attachment energy of a colloidal particle at a fluid–fluid interface [80,81]

(10) $$\Delta E_{Ij}=\Delta F_{Ij}=\Delta E=-\pi R^{2}\gamma_{OW}{\left(1\pm \cos\theta \right)}^{2},$$

where j=O,W and sign “+” (“−”) refers to the transfer from the phase W (O), and, at the same time, it corresponds to the case when the particle centre is located in the phase O (W) [67]. Thus, the particle attachment to the interface is controlled by the particle size and the surface wettability. It is worth noting that ΔE is proportional to $R^{2}$ and rapidly increases with the radii of the nanoparticle.

The particles are attached to the interface if $\Delta E\gg k_{B}T$, where $k_{B}$ is the Boltzmann constant [67]. The attachment (adsorption, trapping) energy, ΔE, increases with the square of the particle radii. The same energy is required for the particle to escape from the interface. The time of the particle desorption, t, depends on the adsorption energy, t∝exp(−ΔE) [1]. The residence time of the nanoparticles increases exponentially with particle size. Thus, the greater particles are much more stable at the interface than the smaller ones. For example, a poly(methyl methacrylate) sphere of radius 50 nm at a hexadecane–water interface needs energy on the order of $10^{5}k_{B}T$ to escape from the interface. This creates a significant barrier, so the particle adsorption practically is irreversible [60]. In general, microparticles are usually irreversibly attached to the interface, while adsorption of nanoparticles at the interface can be tuned by changing the contact angle, θ [67].

#### 2.1.2. Non-Spherical Homogeneous Particles

The shape anisotropy of particles plays a key role in the adsorption of particles on the fluid interface. Non-spherical particles can not only adsorb at different distances from the interface, but they can also orientate in different ways. Therefore, when calculating the attachment energy of such molecules as ellipsoids, cylinders, or dumbbells, one should take into account both the shape and wettability of the particles. The Pierański model [66] can be used also to describe the shape of anisotropic particles. In this case, however, the analytical solutions are difficult, and numerical methods are more appropriate for estimating surface energy [82].

The adsorption energy of non-spherical particles is strongly dependent on their geometries. From Equation (7), it follows that the energy is minimized where the removed area of the interface is maximized. Thus, the particle will have a tendency to lie in the orientation which occupies the greatest interfacial area at the interface [83,84,85]. Ellipsoidal particles adsorb, orienting their major axes parallel to the interface [83]. However, the preferred orientation of cylindrical particles depends on their aspect ratio. The disk (small cylinder height) tends to lie with its base parallel to the interface, while, for very long cylinders, the upright configuration becomes more favorable. In the intermediate cases, both orientations may represent minima in the adsorption free-energy, and a distribution of orientations is observed [86,87,88]. In the case of cubes, there exists an additional rotary axis that generates the possibility of multiple orientations, corresponding to local energy minima in the free energy, and different metastable orientations are observed [89,90,91].

Asdsorption of homogeneous nanorods at fluid–fluid interface was also considered [86,88,92,93]. For the nanorods oriented parallel or perpendicular to the fluid interface, the adsorption energies were calculated. These studies showed that isolated nanorods orient parallelly to the plane of the interface.

In the case of aspherical particles, the effect of line tension is of particular importance, as the contact line is often large relative to the case of a sphere. For example, when an ellipsoid lies flat at the interface, the removed area is greatest, but this orientation also results in a large contact line. Both effects determine the ability of non-spherical particles to retain at the interface.

Faraudo and Bresme [94,95] proposed the thermodynamic model for prolate/oblate particles at interfaces in which line tension was taken into account. In the framework of this model, they discussed the impact of particle geometry and its orientation on stability. The interplay between the geometry and the line tension in determining the stability of nanoparticles at liquid–liquid interfaces was discussed in the work [95]. To understand the effects due solely to the line tension, Faraudo and Bresme [95] assumed that particle–fluid surface tensions are equal. In this case, Equation (5) can be expressed as

(11) $$\Delta F_{I}=\Delta F=-\gamma_{OW}S_{r}+\tau L.$$

The values $S_{r}$ and *L* depend on the shape of the particle and its position at the interface, *d*. Note that a positive line tension destabilizes adsorption at the interface, whereas the liquid–liquid surface tension has the opposite effect, since this reduces the liquid–liquid interfacial area. The relative importance of both effects can be measured using the reduced line tension, defined as

(12) $$\bar{\tau }=\tau {\left(4\pi /S\right)}^{1/2}/\gamma_{OW}.$$

From Equations (11) and (12), as well as the general equation for the free energy of adsorption, can be obtained

(13) $$\Delta F/\left(\gamma_{OW}S\right)=-S_{r}\left(d\right)/S+{\bar{\tau }}_{0}L\left(d\right)/{\left(4\pi S\right)}^{1/2},$$

where ${\bar{\tau }}_{0}$ is a limit value of the reduced line tension [95].

As has been mentioned that the determination of S(d) and L(d) is a difficult task for non-spherical particles. Faraudo and Bresme [95] performed such calculations for spheroids with different aspect ratios in the most stable orientation, which corresponds to the configuration where the shorter axis is perpendicular to the liquid–liquid interface. They derived the explicit expression for the free adsorption energy (Equation (9) in [95]). The analysis of this equation leads to the following conclusions: (i) spheroids with the same total surface area *S*, but different shapes (i.e., different aspect ratios) have different free energies at the interface, and (ii) spheroids with an aspect ratio larger than a certain critical value are not stable at the interface.

**Figure 3:**
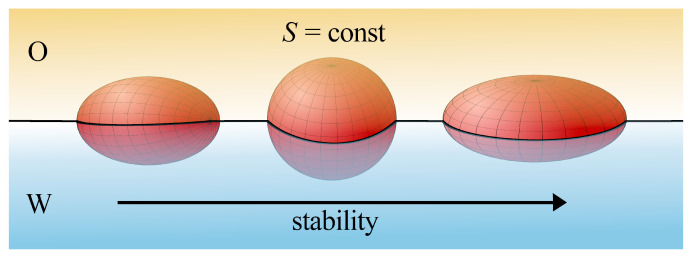
The relative stability of nonspherical nanoparticles at fluid interfaces for particles of different shapes.

When the particles with the same surface area *S* were considered [94,95], oblate particles were stable, the spherical particle would be metastable, while prolate ones would be completely unstable (see Figure 3). Prolate particles (such as nanotubes, viruses, or fibers) are more prone to destabilization due to the line tension than spherical and oblate ones. The theory indicates that the line tension effects are more important for elongated objects. Moreover, in the case of particles with similar volume, the attachment energy changes according to the following order, disks > rods > spheres, and the maximum differences of the energy in relation to that what are found for spherical particles correspond to the extreme wetting conditions ($\theta \approx 0^{\circ }$ and $\theta \approx 180^{\circ }$) [96].

As has been already mentioned, the Pierański-type approaches do not take into account the deformation of the fluid–fluid interface associated with the particle adsorption. These effects were involved in molecular dynamics and lattice-Boltzmann simulations, which led to the results being in general agreement in the phenomenological continuum models [72,97,98,99]. However, Millet and Wang [97] proposed the diffuse-interface field approach to modeling arbitrarily-shaped particles at fluid–fluid interfaces that include external force-induced capillary attraction/repulsion between particles.

Despite all the limitations of the Pierański approach, [66] appeared to be effective in predicting particle configuration at fluid–fluid interfaces. This was supported by molecular simulations performed for different nanoparticles [100,101,102,103].

The existence of different possible configurations of particles at a fluid interface influences the inter-particle interactions, which in turn modifies the assembly of particles at the interface, and consequently they determine the properties of the particle-laden layers.

### 2.2. Self-Assembly of Homogeneous Particles at Fluid–Fluid Interfaces

Fluid–fluid interfaces are deemed as an ideal platform for nanoparticles to self-assemble into high-quality macroscopic monolayer films with low defect density, since they enable the particles to be highly mobile and to rapidly achieve an equilibrium state during self-assembly [2,11,104,105].

To date, a variety of spherical inorganic and organic nanoparticles were applied to create macroscopic monolayer films and superlattices at different liquid–liquid interfaces [3,106,107,108,109,110,111,112,113,114,115,116,117,118,119,120,121,122,123,124,125,126]. The morphology of particle-laden layers was the subject of numerous experimental works. These studies showed that, depending on the interactions between particles and the interfacial coverage, different structures were formed [107,108,109,110,111,112,113,114]. In 1999, Sear et al. [106] presented the results associated with the spontaneous patterning of quantum dots at the water–air interface. Then, the formation of circular and chain-like structures by charged polystyrene spheres at this interface was reported [107]. It was also shown that the change in the particle wettability allows tuning the packing of the structures formed at the fluid interface [108]. For example, the increase in the hydrophobicity of particles caused a transition from loose-packed to close-packed arrays of particles [109]. The correlations between the inter-particle interactions and the different phases emerging in particle-laden interfaces were analyzed [110,111]. Bonales et al. [112] also explored the interfacial organization of mixture monolayers composed of particles having different sizes. Moreover, the experiments proved that increase in the particle density drove the emergence of transitions between phases with a gradually increasing degree of ordering [113,114]. The gas-like, liquid-like films, the hexatic phases, and the highly ordered hexagonal two-dimensional arrays were observed [114].

Recent advances in the synthesis of inorganic nanoparticles have provided nanoscopic objects that are anisotropic in shape. Such particles have attracted great interest in the fabrication of functional materials with novel optical, electrical, and magnetic properties. Wu and coworkers [127,128,129] have developed the self-assembly technique into a novel and facile strategy to fabricate nanofilm-based photodetectors. Hu et al. [127] successfully fabricated the nanofilms by the interfacial assembly of $NiCo_{2}O_{4}$ platelets of a uniform hexagonal shape with sharp corners. These platelets were assembled at a hexane/water interface into a monolayer of high quality. $NiCo_{2}O_{4}$ is a promising material for photodetectors due to its proper bandgap [130]. Biswas and Drzal [131] prepared a monolayer of ultrathin sheets of highly hydrophobic graphene nanosheets at water/chloroform interfaces. However, He et al. [93] studied the self-assembly of tri-n-octyl-phosphine oxide-covered cadmium selenide nanorods at an oil–water interface. It was shown that isolated nanorods lie flat at the interface [93,132]. With increasing nanorod density, the interfacial tension decreases until the interface is saturated with randomly packed nanorods oriented parallel to the interface. Upon further increase in concentration, the separation distance between nanorods decreases to a critical point at which nanorods are forced to reorient normally to the interface to further reduce the energy of the system. The nanorods were assembled into a range of two-dimensional structures with different orientations, from a low-density smectic packing, through a more dense columnar ordering to a crystalline-like phase [93,132]. Valeto et al. [133] proposed a new way to both adsorb and organize microparticles at a liquid interface, with ultralow amounts of a surfactant and no other external forces than gravity. The technique yields different interphase morphologies, from amorphous to highly crystalline two-dimensional assemblies. This results in marked optical properties, such as reflectivity or intense structural coloration.

The experimental studies were supplemented by molecular simulations of particles at fluid–fluid interfaces [101,102,103,106,134]. The experimentally observed assemblies of quantum dots at the water–air interface were reproduced by Monte Carlo simulations [106]. Mowever, Luo et al. [134] used molecular dynamics simulations to investigate the self-assembly of modified hydrocarbon nanoparticles at a water–trichloroethylene interface. The nanoparticles were first distributed randomly in the water phase. The MD simulation showed the formation of nanoparticle clusters and the migration of both single particles and clusters from the water phase to the trichloroethylene phase. Finally, nanoparticles equilibrated in the vicinity of the interface. Striolo and co-workers [101,102,103] compared the behavior of homogeneous and Janus particles at fluid–fluid interfaces using dissipative particle dynamics.

Quite recently, Luo et al. [100] presented results of Monte Carlo simulations of the self-assembly of ellipsoidal particles at fluid–fluid interfaces. They used empirical pair potential that describes the multibody capillary interactions. The simulations well reproduced the experimentally observed [84,135] structures formed by sterically-stabilized ellipsoidal particles onto an oil–air interface at high surface coverage. They found that, depending on aspect ratios and the particle density, in the monolayer, there emerged little clusters, percolating clusters, smectic-like phases, or “raft” structures. At lower surface coverages, it was found that the self-assembly process falls into the diffusion-limited colloid aggregation universality class.

Theoretical models based on continuum thermodynamics are well developed for idealized particle geometries (spheres, ellipsoids, cylinders, etc). However, these models have significant limitations in the presence of microscale features that are much smaller than the particle. Razavi et al. [136] modeled nanoparticles as clusters of spherical atoms and studied the impact of shape imperfections on the particle behavior at the interface. Due to their atomistic nature, these model particles present both microscale and macroscale geometrical features and cannot be accurately modeled as a perfectly smooth body. The molecular dynamics simulations were carried out for the particles with different surface morphologies at the fluid–fluid interface. It was shown that, under certain physical conditions, microscale features could produce free energy barriers that are much larger than the thermal energy of the surrunding media. This effectively “locks” the particle at specific angular orientations with respect to the liquid–liquid interface.

The role of molecular simulations in understanding phenomena emerging at liquid–liquid interfaces was discussed by Razavi, Koplic, and Kretzschmar [137]. They discussed the velocity slip at a liquid–liquid interface, the coalescence of liquid drops in suspension and in free space, and the behavior of colloidal nanoparticles at a liquid–liquid interface. The potential shortcomings and pitfalls of the molecular dynamics simulations were presented.

## 3. Janus Particles at Fluid–Fluid Interfaces

### 3.1. Thermodynamics of Janus Particles at Fluid–Fluid Interfaces

**Figure 4:**
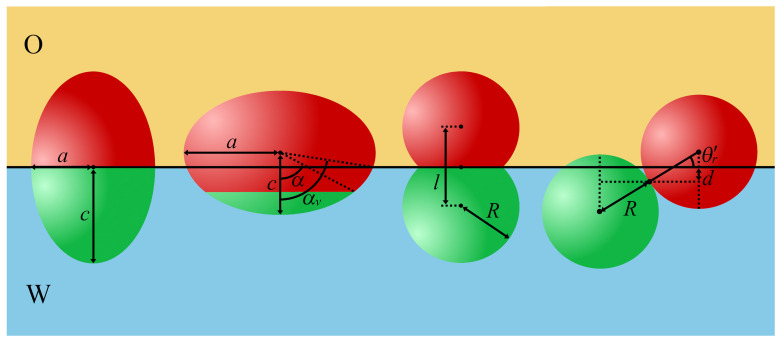
Examples of nonspherical Janus particles at a planar fluid/fluid interface (from left): a symmetrical ellipsoid, an asymmetrical ellipsoid, a symmetrical Janus dumbbell, and a symmetrical Janus dimer. The polar (apolar) regions of Janus particles are green (red). The aspect ratios are defined as follows: $AR_{e}=c/a$ for the ellipsoids and $AR_{d}=\left(2R+l\right)/2R$ for the dumbbells. The angle $\theta_{r}^{\prime }$ characterizes the orientation of the particle with respect to the interface.

The dual nature of Janus particles causes their behavior at the fluid–fluid interface to be very complex. The presence of domains with different wettability creates a new possibility to fine-tune their surface activity. Examples of Janus particles with their geometric characteristics are shown in Figure 2b and Figure 4.

The chemical anisotropy of a Janus particle is characterized by a three-phase contact angle of a polar part ($\theta_{P}$), an apolar compartment with a contact angle ($\theta_{A}$), and the location of the surface boundary partitioning the polar and apolar regions (Janus boundary, wettability separation line). For Janus particles, the degree of amphiphilicity can be defined as $\Delta \theta =\left(\theta_{A}-\theta_{P}\right))/2=\left(\theta_{P}^{\prime }-\theta_{A}^{\prime }\right)/2$. The value $\Delta \theta =0^{\circ }$ corresponds to a homogeneous particle, whereas the maximum possible amphiphilicity is for $\Delta \theta =90^{\circ }$. Moreover, it is useful to formulate the supplementary wettability condition, $\beta^{*}=90^{\circ }-\theta_{A}=\theta_{P}-90^{\circ }$ ($\beta^{*}=90^{\circ }-\theta_{P}^{\prime }=\theta_{A}^{\prime }-90^{\circ }$) [138]. In this case, the two sides of the particle have identical deviations of apolarity and polarity from neutral wetting.

The Pierański-type phenomenological approach can be easily applied to Janus particles. In this case, in Equation (4), the terms associated with the interactions of a particle with both fluids ($S_{O}\gamma_{O}+S_{W}\gamma_{W}$) are replaced by the sum of contributions originating from different surface patches. If the line tension is neglected, we obtain

(14) $$E_{I}=S_{AO}\gamma_{AO}+S_{AW}\gamma_{AW}+S_{PO}\gamma_{PO}+S_{PW}\gamma_{PW}+S_{OW}\gamma_{OW},$$

where $\gamma_{kj}$ and $S_{kj}$ (k=A, *P*; j=O,W) are the surface tension of the kth part of the particle and its surface area in contact with the *j*th fluid, respectively. Moreover, the total surface of the kth patch equals $S_{k}=S_{kW}+S_{kO}$.

From Young’s equations (Equation (4)) for homogeneous polar and apolar spherical particles, we have

(15) $$\cos\theta_{k}=-\cos\theta_{k}^{\prime }=\left(\gamma_{kW}-\gamma_{kO}\right)/\gamma_{OW},$$

where k=P,A.

On the basis of Equations (14) and (15), the attachment energy from the water and oil phase can be expressed as [138]

(16) $$\Delta E_{IW}=\gamma_{OW}\left(S_{AO}\cos\theta_{A}^{\prime }+S_{PO}\cos\theta_{P}^{\prime }-S_{r}\right),$$

(17) $$\Delta E_{IO}=-\gamma_{OW}\left(S_{AW}\cos\theta_{A}^{\prime }+S_{PW}\cos\theta_{P}^{\prime }+S_{r}\right),$$

The trapping energy (Equation (16)) depends on two contact angles and the sizes of both patches on the particle surface. The particle can be differently oriented with respect to the interface. The equilibrium configuration of the particle is characterized by the localization of its center (vertical displacement, *d*) and the parameters associated with orientation. Minimization of the attachment energy (Equation (16) or Equation (17)) with respect to these parameters leads to the expression for the energy at equilibrium.

It should be noted, however, that the areas of a particle immersed in both liquids and the removed area depend strongly on its orientation. It is a bit surprising, but, even for simple shapes of particles, there are no analytical expressions for these areas. For this reason, in many papers, the energy was calculated only for assumed orientations, or numerical methods were used to find these areas [3,60].

### 3.2. Spherical Janus Particles

In this section, we follow the original nomenclature Ondarcuchy et al. [139]. Figure 2b shows that the geometry of the Janus particle is indicated by the angle α, which determines the position of the boundary dividing the apolar (hydrophobic) and polar (hydrophilic) regions on the particle. A given domain is characterized by the contact angle of o a homogeneous particle with the same surface chemical makeup, $\theta_{k}$. The values of $\alpha =0^{\circ }$ or $\alpha =180^{\circ }$ refer to a homogenous particle, whereas $\alpha =90^{\circ }$ corresponds to a symmetrical Janus particle with two equal-sized patches of different wettability. Thus, it is possible to modify the amphiphilicity of Janus particles by changing the wettability of each part or the location of the Janus boundary (α).

Ondarcuchy et al. [139] presented the first theoretical analysis of the behavior of spherical Janus particles at a fluid–fluid interface. They assumed that Janus particles are adsorbed with the wettability separation line parallel to the interface. The rotation of Janus particles is ignored. It was further assumed that the Janus particles are oriented in “the right way” at the interface, that is, with the apolar part directed toward the apolar fluid (O). Then, the total surface free energy ($E_{I}$) for a Janus particle at the interface can be expressed as a function of the angle β that characterizes the immersion depth of the particle, d=Rcosβ (see Figure 4).

(18) $$E_{A}\left(\beta \right)=2\pi R^{2}\left[\gamma_{AO}\left(1+\cos\alpha \right)+\gamma_{PO}\left(\cos\beta -\cos\alpha \right)+\gamma_{PW}\left(1-\cos\beta \right)-0.5\gamma_{OW}\sin^{2}\beta \right],$$

for β≤α

(19) $$E_{P}\left(\beta \right)=2\pi R^{2}\left[\gamma_{AO}\left(1+\cos\beta \right)+\gamma_{AW}\left(\cos\alpha -\cos\beta \right)+\gamma_{PW}\left(1-\cos\alpha \right)-0.5\gamma_{OW}\sin^{2}\beta \right],$$

for β≥α

The energy minimum depends upon the relation between the angles α, $\theta_{A}$, and $\theta_{P}$. One can distinguish three regimes: (i) for $\theta_{P}<\beta <\theta_{A}$ the equilibrium angle β=α, (ii) for $\alpha <\theta_{A}$, $\beta =\theta_{A}$, and (iii) for $\alpha >\theta_{P}$, and $\beta =\theta_{P}$. The first case corresponds to the so-called “Janus behavior” [139]—when a particle is anchored along the Janus line that becomes a contact line between four phases. Thus, each domain is immersed in its preferred fluid phase. If the difference in polarity between the two fluids is significant, the J-behavior dominated in a large range of geometrical asymmetries. However, if $\alpha >\theta_{P}$ or $\alpha <\theta_{A}$, the energy is minimal for either $\theta_{P}$ or $\theta_{A}$. This means that the particle behaves as a homogeneous polar or an apolar one. The Janus boundary is shifted towards the fluid of higher affinity (see Figure 2 in [139]).

Let us focus on particles that present the J-behavior ($\theta_{A}<\alpha <\theta_{P}$). In this case, the adsorption energy from the O-phase (W-phase) is [139,140,141,142]

(20) $$\frac{\Delta E_{IO}}{2\pi R^{2}}=\gamma_{OW}\left[\cos\theta_{P}\left(1-\cos\alpha \right)+0.5\sin^{2}\alpha \right],$$

and

(21) $$\frac{\Delta E_{IW}}{2\pi R^{2}}=\gamma_{OW}\left[-\cos\theta_{A}\left(1+\cos\alpha \right)+0.5\sin^{2}\alpha \right],$$

The adsorption energy is calculated from the expression which gives the lower value. It should be emphasized that the desorption energy of a Janus particle from the bulk fluid ($-\Delta E_{Ij}$) to the interface increases with the particle amphiphilicity. This energy can be several times higher (up to 3-fold higher) than that for a corresponding homogeneous particle [140,143].

In analogy to the hydrophilic–lipophilic balance o and molecular surfactants, Jiang and Granick [141] have proposed the Janus balance defined as the ratio of work to transfer an amphiphilic Janus particle from the oil–water interface to the oil phase, whose work needed to move it to the water phase

(22) $$J=\frac{\sin^{2}\alpha +2\cos\theta_{P}\left(\cos\alpha -1\right)}{\sin^{2}\alpha +2\cos\theta_{A}\left(\cos\alpha +1\right)},$$

If $\theta_{a}$ and $\theta_{P}$ are fixed, the J-balance increases as α increases (larger polar area). An increase in the Janus balance causes a considerable increase in the adsorption energy, and the highest adsorption energy is obtained for J=1 (see Figure 4 in ref. [141]).

Rezvantalab and Shojaei-Zadeh [144] studied the symmetrical Janus particles involving particle rotation and interface deformations. In this case, the part of the polar (apolar) particle can be immersed in the O-fluid (P-fluid). They calculated the attachment energy using the general Equation (16). Each of the particle surface–liquid areas in this formula was estimated numerically through an optimization algorithm that minimizes the total surface free energy. In order to calculate the surface area and interfacial energies at each orientation angle, the simulations were carried out using Surface Evolver [145]. It was assumed that the Janus boundary is perfectly smooth and does not generate interface deformations. In the equilibrium orientation, the apolar and polar hemispheres are fully exposed to oil and water, respectively. In such a case, the particle does not induce any interface deformation, since each region is completely in contact with its preferred liquid phase. In contrast, the particle with tilted orientations has each hemisphere in contact with both liquid phases, and the interface deforms, since each phase tries to wet a larger area of its preferred region of the particle. It was shown that, for non-equilibrium tilted orientations, interface distortion always results in a higher magnitude of the attachment energy. The particle-induced interface deformation results in a rise and depression of the interface around the particle. Furthermore, increasing the particle amphiphilicity results in an increased deviation from the flat interface energies due to a higher extent of interface deformation.

The fluid–fluid interfaces with adsorbed spherical Janus were also studied using molecular simulations. Cheung and Bon [146] investigated the interaction of spherical Janus particles with an ideal fluid interface using Monte Carlo simulations. In the study, the range of the nanoparticle-interface interaction was significantly larger than the nanoparticle radius. In this way, the broadening of the interface due to capillary waves was taken into account. Moreover, in the simulations, the rotation of particles was allowed. The simulation results were compared with those obtained in the framework of continuum theory. For a homogeneous particle, the stability of the particle at a liquid interface decreases as the affinity for one liquid phase is increased relative to the other. In the case of large affinity differences, the detachment energies calculated from continuum theory become increasingly accurate. The simulations showed that the symmetric Janus nanoparticles had a large degree of orientational freedom, in sharp contrast to micrometer-sized colloidal particles. It has been shown that the continuum theory significantly overestimates the detachment energy. This results from the neglect of nanoparticle rotation. Simulations of nanoparticles with fixed orientations showed considerably larger detachment energy. As the areas of the surface regions become asymmetric, the stability of the Janus nanoparticle is decreased, and, in the case of large differences in affinities of the two faces, the difference between detachment energies from simulation and continuum theory diminishes.

Razavi et al. [147] used molecular dynamics simulation to determine the change in Helmholtz free energy, F, of the particle-laden system. The free energy difference between any two points during this transfer process is calculated as:

(23) $$\Delta F\left(z\right)=F\left(z\right)-F\left(z_{ref}\right)=-\int_{z_{ref}}^{z}<f_{z}{>}_{z^{*}}dz^{*}$$

where $<f_{z}{>}_{z^{*}}$ is the ensemble averaged force in the direction normal to the interface acting on the particle at a position $z^{*}$ from the interface. The simulations were performed for plain and Janus particles. The force acting on the particle and the free energy of the system as functions of the particle distance from the interface appeared to be asymmetrical even when the Janus particle is moved quasi-statically. This phenomenon is associated with asymmetric capillary bridging during the adsorption and desorption processes and the formation of a fluid shell surrounding the Janus cap in the desorbed state.

Striolo and co-workers [101,102,103] carried out a series of simulations for Janus particles trapped in fluid–fluid interfaces. Fan et al. [101] used atomistic molecular dynamics simulations to investigate the influence of the surface chemistry of silica nanoparticles on their properties at the water–decane interface. The nanoparticle surface chemistry was changed by systematically varying the surface groups. Methyl groups ($CH_{3}$) represented hydrophobic sites, while hydroxyl groups (OH) were used as representative hydrophilic groups. The overall hydrophobicity of the nanoparticle was characterized by the ratio between $CH_{3}$ and OH groups present at the interface. Nanoparticles with equal overall chemical composition were prepared with different distributions of the various surface groups, leading to homogeneous vs. Janus nanoparticles. The simulations well reproduced values of the experimental values of water/decane surface tensions, the contact angles of the particles, and their configurations at the interface. The impact of the degree of hydrophobicity and the distribution of active sites on the surface on the particle behavior was analyzed. All the nanoparticles pinned to the interface rotated more freely along the plane parallel to the interface than along the plane perpendicular to it.

Dissipative particle dynamics simulations are performed to study the structural and dynamical properties of various systems of spherical nanoparticles accumulated at the water/oil interface [102]. Homogeneous and Janus nanoparticles with different surface compositions are studied. For all nanoparticles, as the surface density increases, a transition from a liquid-like to a solid-like state was observed and at a high density of nanoparticles, and hexagonal structures were found. Mixtures of different spherical nanoparticles were also considered. The same method was used to study the influence of nanoparticles on the water–oil interfacial tension [102].

Jeong et al. [148] applied optical laser tweezers and Monte Carlo simulations to evaluate the effects of the azimuthal rotation of Janus particles at the oil–water interface on interparticle interactions. They found that the capillary-induced attractive force between two Janus particles at the interface can be relaxed by azimuthal rotation around the critical separation region.

### 3.3. Non-Spherical Janus Particles

In the case of non-spherical Janus particles, additional degrees of freedom associated with their rotation can lead to diversity in orientational behaviors when attached to a fluid–fluid interface. The equilibrium configurations of non-spherical Janus particles result from the competition between effects associated with the shape and those following from the chemical anisotropy. First, the particle tends to maximize the surface area removed from the interface, which forces the particles to lie flat on the fluid surface. On the other hand, the anisotropy in wettability leads to the orientation that maximizes interactions with the preferred bulk fluids and can strengthen a tendency to remain in the upright orientation. Different theoretical methods were used to predict the behavior of non-spherical Janus particles, including phenomenological methods, molecular simulation, and density functional theory.

**Figure 5:**
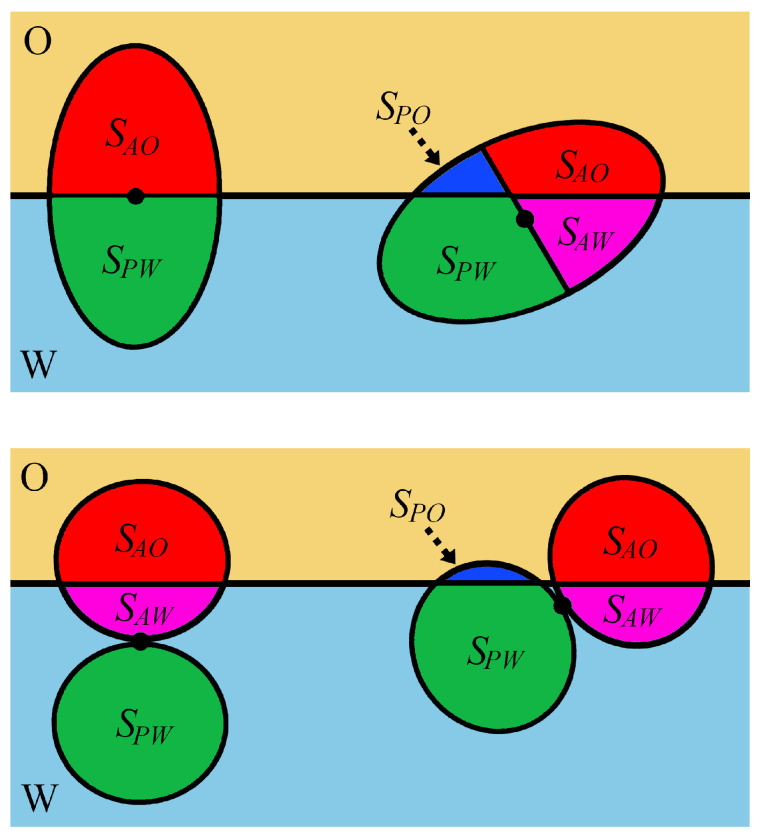
Examples of the configuration of Janus symmetrical ellipsoids (**upper panel**) and symmetrical dimers (**bottom panel**) at a fluid/fluid (O/W) interface. In the case of pinned (unpinned) configuration, the particle center lies at the interface (inside a bulk phase). For an upright configuration, the main axis of the particle is perpendicular to the interface, else a configuration is classified as tilted. Areas of four different particle–fluid interfaces are shown: $S_{PW}$ for a polar region in contact with the fluid W (green), $S_{PO}$ for a polar region in contact with the fluid O (navy), $S_{AO}$ for an apolar region in contact with the fluid O (red), and $S_{AW}$ for an apolar region in contact with the fluid W (magenta).

Examples of studied particles are shown in Figure 4, along with their geometric characteristics. The essence of most methods is the minimization of adsorption energy with regard to parameters characterizing the particle configuration. The adsorption energy was calculated from Equations (16)–(19). To estimate the suitable surface areas, $S_{ij}$ (see Figure 5), various methods were proposed. In the procedure by de Graaf et al. [82], the particle shape is modeled as a series of interconnecting triangles from which the interfacial areas in contact with the liquid phases can be for each particle orientation. Park et al. [138,149,150,151,152] applied a numerical procedure based on the hit-and-miss Monte Carlo method. Moreover, in a number of works, Surface Evolver was utilized [145].

The equilibrium orientations were determined on the basis of a minimum condition in the adsorption energy as a function of the orientation angle with respect to the oil–water interface. In general, non-spherical Janus particles adopt upright or tilted orientations, as shown in Figure 5. In the upright orientation, the long axis of ellipsoids or dumbbells is perpendicular to the interface. In other cases, the particles have tilted orientations. If the major axis is parallel to the interface, the particle adopts a horizontal orientation.

Park and co-workers [138,149,150,151,152] studied the behavior of various non-spherical Janus particles at fluid–fluid interfaces, considering the particles with supplementary wettability. Their approach enables the prediction of the orientation of non-spherical Janus particles and the position of their centers at equilibrium as functions of particle size, aspect ratio, and surface properties. The different configurations of Janus particles at the interface are shown in Figure 5.

Two types of ellipsoids were considered, the symmetrical Janus particles (with the same area of both patches) with the Janus boundary located at the plane perpendicular to the major axis [138] and asymmetrical ellipsoids [149]. In the latter case, the areas of patches A and P were different, and the J-boundary could be perpendicular or parallel to the major axis (see Figure 4) Depending on the value of the aspect ratio ($AR_{e}$), the ellipsoids are prolate ($AR_{e}>1$) [138] or oblate ($AR_{e}<1$) [149]. For the symmetrical and prolate Janus ellipsoids, the center of the particles is located at the fluid–fluid interface, regardless of the values of $\beta^{*}$ and $\theta_{r}$ [138]. It has been shown that a larger difference in the wettability of the two patches and a smaller aspect ratio favors the upright orientation. However, the Janus ellipsoids with a large aspect ratio or a small difference in the wettability of the two regions tend to have a tilted orientation at equilibrium. Another conclusion of this theoretical study is that these particles, under appropriate conditions, can be kinetically trapped in a metastable state due to the presence of a secondary energy minimum. In the next work, Park et al. [149] discussed the pinning and unpinning behaviors of the Janus boundary of chemically anisotropic ellipsoids. They found that the Janus boundary was unpinned when the difference in wettability (b) was not sufficiently high. The particles with large values of α and $AR_{e}$ required stronger wettability to preserve the Janus boundary pinned to the interface.

Similar calculations were carried out for symmetrical Janus dumbbells [138]. These particles adopt the upright orientation if the difference in the wettability of the two sides is large or if the particle aspect ratio is close to 1. Otherwise, the Janus dumbbells would tend to have a tilted orientation at equilibrium. It should be emphasized that Janus dumbbells possess only a primary energy minimum. This indicates that these particles prefer to be in a single orientation. Likely, the thin waist of the Janus dumbbells suppresses the existence of metastable orientations. The absence of multiple minima is potentially advantageous for obtaining particle-laden layers with uniform orientation. Moreover, for Janus ellipsoids and dumbbells, the configurational phase diagrams in the coordinates $AR-\beta^{*}$ were estimated [138,149].

In the case of Janus dimers (AR=1), the equilibrium parameters were determined analytically for $\theta_{A}^{\prime }<90^{\circ }<\theta_{P}^{\prime }$ [153]

(24) $$d_{0}=0.5R\left(\cos\theta_{A}^{\prime }+\cos\theta_{P}^{\prime }\right),$$

(25) $$\sin\theta_{r}^{\prime }=\cos\theta_{r}=0.5\left(\cos\theta_{P}^{\prime }-\cos\theta_{A}^{\prime }\right),$$

where $\theta_{r}$ is the rotation angle describing how the particle axis is oriented relative to the line perpendicular to the interface [150], while $\theta_{r}^{\prime }$ is defined in Figure 4. Indeed, there is only one minimum in the attachment energy. This proves that the Janus dimer does not trap into metastable states at the interface. In the supplementary wettability condition, $\cos\theta_{A}^{\prime }=-\cos\theta_{P}^{\prime }$, so the center of mass of the Janus dimer always is located at the O/W interface ($d_{0}=0$), and the equilibrium orientation angle is $\theta_{r}^{\prime }=\beta^{*}=90^{\circ }-\theta_{P}^{\prime }$. In other words, such particles are pinned at the interface and adopt a tilted orientation for an arbitrary value of $\beta^{*}$, but $\beta^{*}=0$. These theoretical predictions were confirmed by molecular dynamics simulation [153]. Adsorption of Janus dimers at the fluid–fluid interface was also investigated using the density functional theory [154].

Chemically anisotropic cylindrical particles on the fluid–fluid interface were also investigated [151,152,155]. One of the first studies was conducted by Neumann et al. [155], who calculated the adsorption energy of patterned cylindrical particles and stated that the presence of different patches on the particle led to multiple metastable states of particle orientation. Park et al. [151,152] studied experimentally asymmetrically hydrophilic Janus cylinders trapped at an air–water interface. Moreover, they analyzed the configurations of individual Janus cylinders at the air–water interface using the theoretical procedure of minimizing the adsorption energy. It has been shown that the Janus cylinders adopted configurations that have not been previously observed in homogeneous and amphiphilic particles at fluid–fluid interfaces.

The behavior of single non-spherical Janus particles was also studied by means of molecular simulations [153,156,157,158]. We discuss, here, a few representative examples.

Gao et al. [156] used molecular dynamics simulations to study the influence of the shape of JPs on their orientation and interface activity at fluid–fluid interfaces. The Janus particles were constructed by lumping Lennard-Jones (LJ) beads with different types at one of the two halves of the Janus partricle surface. Three types of Janus particles were considered: spheres, “rods”, and discs. The “rods” are ellipsoids with the J-boundary along a major axis, while the discs have different top and bottom sides. The free energy profiles for these particles transferring from a fluid phase to the interface were calculated. Different initial orientations of the particles with respect to the interface were considered. For Janus spheres and rods, the free energy achieves a minimum at the interface, irrespective of the initial orientation of the JPs. Therefore, there is one equilibrium orientation for both Janus spheres and Janus rods at the fluid–fluid interface. In contrast, Janus discs with different initial orientations follow different pathways, ending up in two different final orientations, corresponding to forward orientation and reverse orientation. Thus, some Janus discs can be kinetically trapped in a metastable orientation, and the initial orientations of Janus discs have a great impact on their final orientation. The attachment free energies satisfy the relation sphere > rod > disc. The Janus discs are the most efficient to stabilize a fluid interface. However, this interface may be metastable, depending on the orientation of the Janus discs at the interface.

The impact of Janus particles on the fluid–fluid interfacial tension was also studied [156]. The reduction of the interfacial tension depends on the particle shape. The Janus spheres are the weakest in reducing surface tension, whereas Janus rods are the most efficient. Moreover, the time evolution of interfacial tension of the fluid–fluid interface by adsorbing the different Janus particles was analyzed. The interfacial tension showed a rapid decrease at the early stages of adsorption for all types of Janus particles, which leveled off and reached a stable value. The time evolution of interfacial tension could be associated with the multiple orientations and the slow time scale for reorientation at the interface. Spheres were observed to equilibrate rapidly. In contrast, Janus discs, which have two energy minima, and therefore must reorientate after initial adsorption, require a long time to reach equilibrium.

Striolo and coworkers [157,158] used dissipative particle dynamics simulations to investigate the ellipsoidal Janus nanoparticles adsorbed at flat and spherical oil/water interfaces. The studies showed that the Janus ellipsoid has one preferential orientation but oscillates around it [157]. At a sufficiently high surface coverage, the nanoparticles are most effective at reducing the interfacial tension when they lay with their longest axis parallel to the interface. It was found that the orientation of the nanoparticles with respect to the interface depends on nanoparticle aspect ratio, on the amount of polar and nonpolar surface groups, and on the interactions between the nanoparticles’ surface groups and aqueous and non-aqueous solvents. Analysis of simulation results suggested that prolate and oblate nanoparticles are more effective than spherical particles in reducing interfacial tension [156]. These results are in reasonable agreement with the experimental data of interface activity measured by Ruhland et al. [159,160].

Anzivino et al. [161] investigated the adsorption of a variety of Janus particles (dumbbells, elongated dumbbells, and spherocylinders) at a fluid–fluid interface by using a numerical method that takes into account the interfacial deformations. They found that the overall shape of the induced deformation field has a strong hexapolar mode for individual Janus particles, while non-Janus particles of the same shape do not induce any interfacial deformation. It was shown that the hexapolar deformation field induces capillary attractions for laterally aligned Janus sphero-cylinders and repulsions for laterally anti-aligned ones. The theoretical predictions were confirmed experimentally.

Quite recently, Gunther et al. [162] presented a simplified free energy model of an ellipsoidal Janus particle at a non-deforming fluid–fluid interface and then extended it by taking into account the interface deformation. The theoretical models were used to determine the equilibrium orientation of the particle. These predictions were compared with the results obtained from lattice Boltzmann simulations. The studies showed that, at equilibrium, the Janus ellipsoid had a tilted orientation for large aspect ratios and small wettability differences, where the shape dominates. However, for small aspect ratios and large wettability differences, the Janus effects dominated, and the particle was in the upright orientation.

Hossain et al. [163] used dissipative particle dynamics simulation to investigate the translational diffusion of Janus rods at the interface between two immiscible fluids. They showed that the particle aspect ratio affects both particle’s translational thermal motion and the average orientation of the particle with respect to the interface at equilibrium. This work provides a deep insight into the dynamics and self-assembly of anisotropic Brownian particles at interfaces.

### 3.4. Self-Assembly of Janus Particles at Fluid–Fluid Interfaces

Particle–particle interactions have a direct impact on the assembly of Janus particles at fluid–fluid interfaces. They depend on the particle size, the chemical nature of particle patches, and the fluid and interface deformations. It was shown that contact line pinning on Janus particles induces irregular interface deformations, leading to attractive interactions [164,165]. Moreover, the orientation of adsorbed particles with respect to the interface affects both the energy landscape of isolated particles and the particle pairs [144]. For two particles at different orientations, decreasing the interparticle distance caused the overlap of interface distortions, producing strong capillary interactions. When the orientation angles are similar, interface deformations do not overlap, and the total surface energy increased as the particles approached one another, which leads to repulsive forces between the particles. However, when particles with opposite orientation angles approached each other, the surface energy decreased, inducing attractive forces to form capillary bridges [87]. The overall shape of the interface depends on distortions caused by neighboring particles. Particle–particle interactions at fluid interfaces are highly dependent on particle shape [150,151,152]. Tilted Janus cylinders at the interface were shown to form side-to-side and tail-to-head orientations due to quasi-quadrupolar interface deformations [152]. Double hydrophilic Janus cylinders (two sides have different degrees of hydrophilicity) induced asymmetric hexapolar interface deformations, resulting in strong lateral attractive interactions forming diverse assemblies [150].

A complex interplay between different interactions at the interface shapes its morphology. A lot of nanoparticle structures were observed in experiments [144,164,165,166,167,168,169]. For example, Park et al. [164] studied the aggregation of Janus particles at the fluid–fluid interface and reported the formation of fractal-like clusters. They proved that the quadrupolar capillary interactions between spherical Janus particles account for the observed aggregation of the particles. The capillary forces were generated by the irregular shape of the Janus boundary, which is intrinsic to the Janus particle fabrication. Moreover, it was reported that two-dimensional films of Au-coated Janus particles and homogeneous particles at air–water interfaces showed different responses under compressive stresses [166]. The relatively weak capillary interactions on the homogeneous particles allowed them to form a densely packed colloidal monolayer membrane that withstands large compression and forms wrinkles. In this case, the particle films underwent a transition from wrinkling to out-of-plane fold deformations. The Janus particles form films with random orientations and complex contact lines. This generates strong, non-uniform capillary interactions, which lead to subduction behavior when particles slip underneath one another during compression. The Janus particles can also form hexagonally packed assemblies at fluid–fluid interfaces that can be transferred to solid substrates for various applications [167]. The self-assembly in ternary systems involving oil, water, and amphiphilic Janus particles was experimentally studied [168]. With increasing water content, the self-assembled structures showed a transition from small micelle-like clusters, through rod-shape clusters, to emulsions, where spherical droplets were stabilized by the surface Janus particles adsorbed at the interface. It was also demonstrated that the chemical duality of the particles induces self-assembly, which is qualitatively the same as that in microemulsions of surfactant molecules. There is, however, an essential difference: the mesoscopic size of the particles makes the interparticle and particle-interface interaction much larger than thermal agitation. The self-assembly in the system involving Janus particles is therefore irreversible, and the structures are kinetically, not thermodynamically, stabilized. Interesting results were reported by Kozina et al. [169]. They found that mixtures of amphiphilic Janus and homogeneous hydrophobic particles formed the bilayer structures at an air/water interface. Despite their strong interfacial adsorption, Janus particles formed the slightly shifted upper layer.

To give a deeper insight into the mechanism of self-assembly of Janus particles trapped at fluid–fluid interfaces, molecular simulations were performed. Gao et al. [156] analyzed the adsorption of Janus “rods” at the fluid–fluid interface. In the bulk fluids, the particles formed linear aggregates. The was characterized by three adsorption stages: free diffusion of some particles to the interface, the adsorption of Janus particles to form domains at the interface, and particle packing with rearrangement into orientationally ordered domains. Lu et al. [157] studied the behavior of ellipsoidal Janus nanoparticles with different shapes, surface chemistry, and density at the water–oil interface using dissipative particle dynamics simulations. The averaged orientation of a particle with respect to the interface was discussed. In the case of the spherical Janus particle, the average orientation did not change when either the particle surface properties or the particle surface density were changed. For ellipsoidal particles, both factors affected the averaged nanoparticle orientation. As the aspect ratio was high, isotropic-to-nematic phase transition was observed. However, only in some cases, it was found that increasing the particle surface density affected the average orientation. For a prolate particle with the highest considered aspect ratio, the simulation results provided evidence for an isotropic-to-nematic phase transition. This transition seems to occur without significant changes in the average particle orientation with respect to the liquid–liquid interface. Similar simulations were carried out for ellipsoidal Janus nanoparticles adsorbed at spherical oil/water interfaces [158]. The nanoparticles were found to yield isotropic, radial nematic phases, and axial nematic domains, depending on the nanoparticle characteristics, particle density at the interface, and droplet properties. When adsorbed on water droplets, the nanoparticles with a high aspect ratio and few nonpolar beads on their surface could show two preferred orientation angles. In contrast, only one equilibrium orientation was found for such nanoparticles adsorbed on oil droplets. Recently, Striolo et al. [170] have used DPD simulations to study the self-assembly of nanoparticles on liquid crystal (LC), and oil–water nano-droplets were investigated. Under the conditions chosen, the LCs formed bipolar nano-droplets. Adsorption of homogeneous and Janus nanoparticles of various geometrical shapes on these droplets was studied. These simulations suggested a complex interplay between nanoparticle size, shape, and chemical properties and their self-assembly on LC nano-droplets.

These examples illustrate the power of molecular simulations as a tool for elucidating the microscopic details of a system invisible to most experimental tools.

## 4. Hairy Particles at Fluid–Fluid Interfaces

### 4.1. Individual Hairy Particles at Fluid–Fluid Interfaces

The behavior of nanoparticles at fluid–fluid interfaces can be changed by modification of their surfaces with various ligands, for example, polymers of different internal structures and chemistry, liquid crystals, proteins, etc. [56,57,171]. In this way, we can tune the particle properties in a wide range. This, in turn, allows us to control their adsorption at the interfaces, the stability of the particle-laden layers, and their morphology.

The ligand-tethered particles are the subject of numerous studies summarized in several reviews [56,57,172,173]. The theoretical research involved scaling theories, self-consistent field methods, and computer simulations. Most of the research focused on the morphology of polymer coatings by changing ligand properties, the grafting density, the interactions of chains with the environment, and the temperature. Ohno et al. [174,175] extended the mean-field theory of star polymers [176] to the polymer-tethered spherical particles of different sizes. The self-consistent field model and the scaling theory were also used to study configurations of chains tethered on spherical particles [177,178]. Lo Verso et al. [179] applied density functional theory to study polymers end-grafted to spherical nanoparticles under good solvent conditions. In turn, Ginzburg [180] used a self-consitent field–density functional theory approach to show that neat hairy particles form lamellar, cylindrical, and spherical phases for various nanoparticle volume fractions. Readers interested in the theoretical problems associated with polymer brushes are referred to the comprehensive review [172,173].

The properties of polymer canopies were widely explored using fully atomistic molecular simulations [181,182,183,184,185,186,187,188]. Among others, the reorganization of ligands tethered to nanoparticles under different environmental conditions was studied [189,190,191]. Dong and Zhou [189] performed coarse-grained simulations for particles modified with block copolymers or mixed polymer brushes to investigate their responsive behavior in different solvents. Depending on the nature of polymer coatings and solvents, different structures were found: typical core-shell, Janus-type, buckle-like, ring-like, jellyfish-like, and octopus-like morphologies. In some cases, the reconfiguration inside the polymer shell can cause the formation of “patchy” nanoparticles [190]. Staszewski [191] studied the behavior of mobile ligands on the surface of nanospheres and analyzed the influence of the type of ligands, their number, and the strength of interactions on the structure of the polymer layers. Similar simulations were used to study the behavior of polymer ethered particles immersed in fluids of isotropic particles [192,193]. It was shown that adsorption of isotropic particles “on chains” caused reconfiguration of the tethered chains, leading to a variety of morphologies, including typical core-shell structures and octopus-like and corn-like structures [192]. The impact of the solid surface on the configurations of tethered polymers was also analyzed [194,195]. Furthermore, numerous studies have focused on the self-assembly of hairy particles in bulk systems, as these particles are promising building blocks for the production of novel nanocomposites [196,197,198]. The new structures were found in the two-dimensional systems that, to some degree, mimic the interfacial systems [199,200].

A lot of research has been also done on the properties of the liquid–liquid interface, involving hairy particles [108,109,125,182,201,202,203,204,205,206,207,208,209,210,211,212,213,214,215,216,217,218,219,220,221,222,223,224,225,226,227,228,229,230,231,232,233,234,235,236,237]. The surface modification changes the contact angle of particles and their adsorption at the interface. Depending on the nature of attached ligands, the interaction of the particle with a fluid interface can vary from repulsive to attractive. A common method to continuously tune the contact angle of particles is imparting different degrees of hydrophobicity by introducing a different number of hydrophobic groups to the surface [201]. Polar functional groups, especially charged end groups, embedded in ligands, strongly affect interactions of the particle with the interface. Electrostatic effects at the liquid–liquid interface can prevent particle adsorption [202] or enhance it [203,204]. As a consequence, the conditions for stability in the bulk suspension can be antagonistic to the conditions for adsorption to fluid–fluid interfaces [58]. Another significant factor that decides the interfacial properties of hairy particles is the grafting density. In dense layers, the tehthered polymers assume a more extended configuration. However, a sparse polymer layer allows a polar liquid to penetrate, causing the collapse [205]. The nature of the grafting bond also plays a considerable role, for example, the thiol–gold bond is relatively mobile, allowing thiolated ligands to move along the nanoparticle surface, while polyelectrolyte brushes grown from the surface of silica particles are irreversibly attached [206]. Moreover, ligands can be linked to the core via covalent bonds, then a well controlled polymer layer is formed with a fixed number of tethers in a particle. However, various molecules can be also reversibly adsorbed at the particle core, and a number of such „ligands” varies with the concentration in the system.

The addition of different chemical additives to the particle dispersion is the most widespread strategy to modify the ability of particles to remain trapped at the fluid interface. One of the most popular additives are surfactants that can screen or suppress the particle surface charge [207,208], change the particle wettability [209,210], or modify their ability the stabilization of emulsions and foams [109,211,212].

An important feature of nanoparticles is that the length of grafted ligands is usually comparable to the size of the core. Therefore, ligand configuration and rearrangements can dramatically affect nanoparticle interactions in the bulk fluid, as well as interparticle interactions at fluid interfaces. In contrast to the particles with a “fixed” internal structure, hairy particles change their configurations in response to the surrounding environment and temperature. If the temperature varies, the tethered chains can form coils or become more stretched [172,179]. As a result, the shape of the particle changes, which in turn affects its ability to be trapped in the interface. Thus, the temperature is the important parameter controlling the adsorption of hairy particles on fluid–fluid interfaces [62].

In this section, we briefly described the impact of such changes on the behavior of individual hairy particles on the fluid–fluid interfaces. When a particle straddles an air–water or oil–water interface, the ligands on the two sides of the interface can adopt different configurations depending on the nature of the ligands, the solvent quality, the grafting density, and other parameters [58]. Theoretical studies were carried out in parallel with experimental research [189,213,214,215,216,217,218]. The impact of the trapping at the fluid–fluid interface on the morphology of individual hairy particles was studied by molecular simulation [189]. Quan et al. [213] studied the structural properties of amphiphilic polymer-brush-grafted gold nanoparticles at the oil–water interface, which were investigated by coarse-grained simulations. The effects of grafting architecture (diblock, mixed, and Janus brush-grafted particles ) and hydrophilicity of polymer brushes were discussed. The simulation results indicated that functionalized gold nanoparticles presented abundant morphologies, including typical core-shell, Janus-type, jellyfish-like, etc., in a water or oil-water solvent environment. The results demonstrated that the Janus brush-grafted particles had the highest interfacial stability and activity, which can be further strengthened by increasing the hydrophilicity of grafted ligands. Tang et al. [214] used molecular dynamics simulation to study hairy particles in a polymer bilayer formed by two mutually immiscible polymers. They showed how the grafting density and interactions with the free polymers affect the free energy profiles. They found that these profiles are quadratic functions of the distance from the interface, as it is predicted for hard particles (see Equation (8)).

Molecular dynamics simulations also revealed that the free energy of rearrangement of the ligands contributes to the total change in free energy upon adsorption of the particle. This complicates the simple picture that treats nanoparticles as rigid spheres with a well defined contact angle with the two fluid phases. Nevertheless, the experimental values of ΔF [215,216] agree in order of magnitude with those obtained from Equation (1). Tay and Bresme [217] used molecular dynamics simulations to determine the contact angles of alkylthiol-passivated gold nanocrystals adsorbed at the air–water interface. They demonstrated that the length of the surfactant chain profoundly affected the wetting behavior of these nanoparticles. The simulations showed that the shape of the hairy particles was strongly perturbed by the interface.

**Figure 6:**
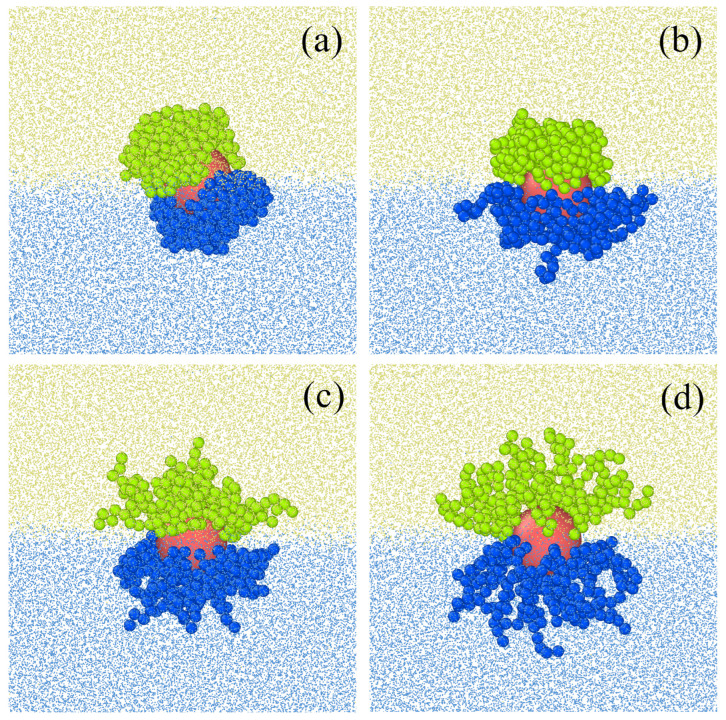
Examples of equilibrium configurations of Janus hairy particles obtained from molecular dynamics simulations. The Lennard-Jones parameters characterizing interactions of chains A (navy) with the fluid W, $\varepsilon_{AW}^{*}$ and chains B (green) with the fluid W, $\varepsilon_{BO}^{*}$: (**a**) $\varepsilon_{AW}^{*}=\varepsilon_{BO}^{*}=0.5$, (**b**) $\varepsilon_{AW}^{*}=0.5$ and $\varepsilon_{BO}^{*}=1$, (**c**) $\varepsilon_{AW}^{*}=\varepsilon_{BO}^{*}=1$, and (**d**) $\varepsilon_{AW}^{*}=\varepsilon_{BO}^{*}=1.5$. Other interactions with chains are assumed to be repulsive.

Figure 6 shows equilibrium configurations of Janus-like hairy particles at the liquid–liquid interface obtained from molecular dynamic simulations. The interface and the hairy particles were modelled according with procedures described in works [153,194,195]. Interactions between “atoms” are modeled by the shifted-force Jenard-Jones potentials. All segment interactions, except interactions with a preferred liquid, are assumed to be repulsive. The segments A (navy) atract molecules of the fluid W, while segments B (green) attract molecules of the fluid O. We see that a change o of these intercations affects considerably the particle shape, size, and symmetry.

A significant role of particle reconfiguration was confirmed by DPD simulations of Brownian diffusion of Janus nanoparticles at water–oil interfaces [218]. Their diffusion was found to be significantly slower than that of homogeneous nanoparticles. However, a good agreement between experimental and simulation results was obtained only when the flexibility of particle shape had been taken into account. The polymeric ligands were deformed and oriented at an interface so that the effective radius of Janus nanoparticles is larger than the nominal one obtained by measuring the diffusion in bulk solution.

### 4.2. Self-Assembly of Hairy Particles at Fluid–Fluid Interfaces

The attachment to nanoparticle ligands can change inter-particle interactions, which in turn determine the final structure of the assembled particle-laden interface. The assembly nanoparticles with chemically bonded ligands were studied experimentally. We begin with a brief presentation of a few representative experimental results. Park et al. [125] investigated “bare” gold and hairy nanoparticles at the water/hexane interface. In the first case, the monolayers had a disordered structure. However, the particles with linked 1-dodecanothiol molecules formed a close-packed monolayer with a high degree of order. The ligands caused a decrease in the surface charge density of the particles, which minimizes the unfavorable electrostatic repulsions and favors the ordering of the particles within the interface. Kim et al. [219] found that the increase in the length of the hydrophobic ligands allowed passing from ordered liquid two-dimensional phases to a disordered two-dimensional solid one. The mechanism of the structural transitions was discussed. The impact of the length of the tethered chain on the assembly of hairy particles at the fluid interface was also explored by Isa et al. [220]. Furthermore, the effect of the reversible adsorption of additives on the nanoparticle surface on the structure of the interfacial layer was the subject of numerous experimental works [108]. As has already been mentioned, the use of the surfactant-decorated particles allowed control of the adsorption and assembly of the particles at the fluid interface [108,221,222,223]. In this way, it is possible to reduce the strength of the repulsive electrostatic interaction, fostering colloidal aggregation, which limits the formation of structures with long-range positional order [221]. Velikov et al. [224] obtained long-range two-dimensional crystals by capping particles with a surfactant at a concentration that ensures particle hydrophobization that hindered bulk aggregation. It was shown that by tuning the ratio between the concentrations of surfactant and nanoparticles, it is possible to obtain different disordered and two-dimensional crystal phases [225,226].

The assembly of hairy particles at fluid–fluid interfaces was studied by molecular simulation [182,215,227,228,229,230,231,232,233,234,235,236,237]. Grest and coworkers carried out classical atomistic molecular simulations of hairy particles in solutions [182,183] at the liquid–vapor interface [182,227,228] and at membranes [229]. They observed a spontaneous asymmetry of spherical hairy particles in solutions and at the liquid–vapor interface. These asymmetric and oriented coatings affect the interactions between nanoparticles and their self-assembly [182]. They showed how the nanoparticle assembly can be controlled by using different ligands. The particle exhibited environment-responsive shapes. The simulations [228] showed that varying the terminal groups of a nanoparticle canopy strongly alters the coating shape at the water liquid–vapor interface, which leads to different assembly morphologies (short linear clusters with a highly aligned structure, dimers, and disordered clumps). The structure and mechanical properties of membranes built of hairy particles were also analyzed [229,230]. Gupta and Escobedo [231] used non-equilibrium molecular dynamics simulations to understand the driving forces behind the formation of highly ordered, epitaxially connected superlattices of polyhedral-shaped nanoparticles at fluid–fluid interfaces. It was shown that differences in nanoparticle shapes and time-dependent facet-specific ligand densities cause different transformation mechanisms. These results indicate that the inter-particle interactions by the surrounding solvation environment have a significant impact on reversibility and ultimately the coherence of the final two-dimensional superlattice obtained. For the particle shapes examined, a hexagonal pre-assembly and a square superlattice final assembly were prevalent. However, depending on the particle shape, different structures can be also formed. Tang et al. [214] presented the molecular dynamics study of the assembly of spherical hairy nanoparticles at the polymer–polymer interface. Depending on assumed parameters, the particles formed a variety of unusual nanostructures, such as dimers with tunable tilt relative to the interface, trimers with tunable bending angle, and anisotropic macroscopic phases, including serpentine and branched structures, ridged hexagonal monolayers, and square-ordered bilayers. In their next work [232], the self-assembly of hairy nanocubes at the polymer–polymer interface was studied. They showed that the nanocubes can be induced into face-up, edge-up, or vertex-up orientations by tuning the graft density and differences in their miscibility with the two polymer layers. The resulting orientationally constrained nanocubes were assembled into a variety of unusual architectures, such as rectilinear strings, close-packed sheets, bilayer ribbons, and perforated sheets.

Tailoring interfacial organization of hairy Janus nanoparticles through entropy was studied using DPD simulations [215,233,234,235,236,237]. The impact of molecular architectures on the entropic contributions to interfacial self-assembly was summarized in the review [233]. The entropy-mediated nanoparticle organization arising from the collective behavior of binary mixtures of hairy Janus nanoparticles at the fluid–fluid interface was discussed [234]. Through applying mechanical pressure, the system exhibits a mechanical response characterized by the reversible transition between the random state and the long-ranged intercalation state of different nanoparticle components. It was also found that the entropy-templated assembly in binary mixtures of hairy nanoparticles at the liquid–liquid interface significantly depends on the stiffness of tethered chains [235]. The considered systems evolved into binary nanoparticle superlattices by remixing at extremely high stiffness. The effects of the polymerization reaction initiated from the surfaces of just one component of nanoparticles were considered [236]. The simulations demonstrated that the competition between the reaction rate and the diffusive dynamics of nanoparticles governed the implementation of entropy in driving the phase transition from randomly mixed phase to intercalated phase in these interfacial nanoparticle mixtures. Furthermore, the entropy-driven self-assembly of hairy Janus particles at a sphere was also studied. By combining large-scale molecular simulations and theoretical analysis, it was demonstrated that the interplay among anisotropy, curvature, and chain conformation induces these particles to assemble into diverse novel structures [237]. Depending on the length asymmetry of tethered chains and Janus balance of the nanoparticles, different aggregates are observed, including binary nanocluster, trinary nanocluster, nanoribbon, and hexagon with centered reverse.

The information that one can extract from experimental studies of complex systems is limited and molecular simulations complement them and help their interpretation.

## 5. Concluding Remarks

In this article, we have briefly reviewed the theoretical studies of Janus particles and ligand-tethered particles at liquid–liquid interfaces. The most representative articles devoted to the discussed issues are summarized in Table 1. We have focused on the extension of the phenomenological proposed by Pierański [167] for Janus particles and molecular simulations. In these approaches, the particles are treated as hard objects. However, in the case of hairy particles, we emphasize the flexibility of their organic canopy and the reconfiguration at the interface.

First, we have presented the thermodynamic foundations of equilibrium at a fluid–fluid interface, involving individual particles with smooth, homogeneous surfaces. Next, we have described a general method for the calculation of the attachment energy of Janus particles of different shapes. Using the obtained expressions, we have discussed the impact of different parameters on the adsorption of particles at fluid–fluid interfaces. In general, the conditions for spontaneous adsorption at an interface are dictated by the balance of interfacial energies between the particle and the two fluids. The principal driving force for nanoparticle adsorption is the removal of an area of fluid interface of high interfacial energy when the nanoparticle moves from the bulk phase to the interface. The adsorption of homogeneous particles depends on their shape and wettability. In the case of Janus particles, the amphiphilicity of the particle also plays an important role, and so does the distribution of different patches at its surface. This can significantly increase the surface activity of the particle and expands the possibilities of controlling its properties. Non-spherical particles can be differently oriented to the interface. In this case, the free energy landscape is usually very complicated, and meta-stable orientations are observed. The molecular simulations referenced in this review proved that the phenomenological models quite well describe the most important properties of the particles at the interface. On the other hand, the simulations give deeper insight into the relations between interactions in the system and the behavior of individual particles at the fluid–fluid interface.

The interactions between adsorbed particles affect their assembly at the fluid–fluid interface. As has been described in Section 2.2, Section 3.4 and Section 4.2, the interfacial films with different degrees of the order are observed in experiments. These structures are reproduced by numerous simulations. We have also highlighted open issues in understanding the thermodynamic properties of interfacial monolayers of nanoparticles.

The goal of this review was to develop the link between phenomenological models of particles at fluid–fluid interfaces and molecular simulations. The simple model is surprisingly effective in predicting the behavior of various particles at such interfaces. Their main advantage is that they can be easily used in practice. On the other hand, these models omit important factors affecting the interfacial equilibrium. Molecular simulations develop significant information on the morphology of particle-laden layers, for example, a type of translational and orientational ordering, the distribution of particle orientations, the shape of ligand-tethered particles, the free energy profiles, and many others. Once these connections are established, we can more efficiently design particle-filled layers to suit a variety of applications.

A deeper understanding of the behavior of hybrid nanoparticles at interfaces will help theoretical studies and their practical usage in nanotechnology.

## Figures and Tables

**Table 1:** The discussed issues with the relevant references.

Fluid–Fluid Interfaces:	
Issues under Consideration	Representative References
Phenomenological models:	
- homogeneous particles	[1,2,3,4,5,6,60,67,78,79,80,81]
- Janus particles	[138,139,140,141,143,144,146,147,149,150,151,152]
Attachement energy:	
- homogeneous particles	[1,2,3,4,5,6,60,67,78,79,80,81,94,95]
- Janus particles	[138,139,140,141,143,144,146,147,149,150,151,152]
Line tension effects	[1,60,65,68,69,71]
Particle shape effects	[83,84,85,86,87,88,89,90,91,92,93,94,95]
Capillary effects	[3,60,72,73,148]
Partricle roughness effects	[60,74,75,76]
Self-assembly of homogeneous particles	
- experiments	[2,3,93,106,107,108,109,110,111,112,113,114,115,116,117,118,119,120,121,122,123,124,125,126,127,128,129,130,131,132,133]
- simulations	[100,101,102,103,106,134,136,137]
Molecular simulations of Janus particles	[153,156,157,158,160,161,162,163]
Self-assembly of Janus particles	
- experiments	[144,164,165,166,167,168,169]
- simulations	[156,157,158,170]
Molecular simulations of hairy particles	[189,213,214,215,216,217,218]
Self-assembly of hairy particles	
- experiments	[108,125,219,220,221,222,223,224,225,226]
- simulations	[182,215,227,228,229,230,231,232,233,234,235,236,237]
Application of hybrid particles	[7,8,9,10,11,13,14,27,28,29,30,31,32,33,34,35,36,37,38,39,40,41,42,43,44,45,46,47,48,49,50,51,52,53,54,55,56,57,58,58]
Relevant reviews	[1,2,3,4,5,6,10,11,13,14,54,55,59,60,61]

## Data Availability

Not applicable.
